# Using Serology to Assist with Complicated Post-Exposure Prophylaxis for Rabies and Australian Bat Lyssavirus

**DOI:** 10.1371/journal.pntd.0002066

**Published:** 2013-02-28

**Authors:** Niall Conroy, Susan Vlack, Julian M. Williams, John J. Patten, Robert L. Horvath, Stephen B. Lambert

**Affiliations:** 1 Public Health Registrar, Queensland Health, Brisbane, Queensland, Australia; 2 Moreton Bay Public Health Unit, Queensland Health, Redcliffe, Queensland, Australia; 3 Royal Brisbane and Women's Hospital, Queensland Health, Brisbane, Queensland, Australia; 4 Brisbane Sexual Health Clinic & AIDS Medical Unit, Queensland Health, Brisbane, Queensland, Australia; 5 Prince Charles Hospital, Queensland Health, Chermside, Queensland, Australia; 6 Queensland Children's Medical Research Institute, The University of Queensland and Queensland Children's Health Services, Queensland Health, Brisbane, Queensland, Australia, and Immunisation Program, Communicable Diseases Branch, Queensland Health, Brisbane, Queensland, Australia; Centers for Disease Control and Prevention, United States of America

## Abstract

**Background:**

Australia uses a protocol combining human rabies immunoglobulin (HRIG) and rabies vaccine for post-exposure prophylaxis (PEP) of rabies and Australian bat lyssavirus (ABLV), with the aim of achieving an antibody titre of ≥0.5 IU/ml, as per World Health Organization (WHO) guidelines, as soon as possible.

**Methodology/Principal Findings:**

We present the course of PEP administration and serological testing for four men with complex requirements. Following dog bites in Thailand, two men (62 years old, 25 years old) received no HRIG and had delayed vaccine courses: 23 days between dose two and three, and 18 days between dose one and two, respectively. Both seroconverted following dose four. Another 62-year-old male, who was HIV-positive (normal CD4 count), also suffered a dog bite and had delayed care receiving IM rabies vaccine on days six and nine in Thailand. Back in Australia, he received three single and one double dose IM vaccines followed by another double dose of vaccine, delivered intradermally and subcutaneously, before seroconverting. A 23-year-old male with a history of allergies received simultaneous HRIG and vaccine following potential ABLV exposure, and developed rash, facial oedema and throat tingling, which was treated with a parenteral antihistamine and tapering dose of steroids. Serology showed he seroconverted following dose four.

**Conclusions/Significance:**

These cases show that PEP can be complicated by exposures in tourist settings where reliable prophylaxis may not be available, where treatment is delayed or deviates from World Health Organization recommendations. Due to the potentially short incubation time of rabies/ABLV, timely prophylaxis after a potential exposure is needed to ensure a prompt and adequate immune response, particularly in patients who are immune-suppressed or who have not received HRIG. Serology should be used to confirm an adequate response to PEP when treatment is delayed or where a concurrent immunosuppressing medical condition or therapy exists.

## Author Summary

In Australia, the administration of rabies post-exposure prophylaxis (PEP) occurs for potentially exposed returned travellers from endemic regions or for potential local exposure to Australian Bat Lyssavirus. For Australian tourists, delays in commencing PEP or not receiving HRIG or all recommended doses of vaccine are common. We report a case series where serology provided information in four patients with delayed, incomplete, or complicated PEP treatment. Three of these patients reported a dog bite in Thailand and the fourth was scratched by a bat and had bat urine enter his eye in Australia. Management was complicated by lack of HRIG administration, delays in the recommended timeframe for receipt of vaccine doses, and immunosuppression caused by co-administration of steroids and by HIV infection with a normal CD4 count. All patients seroconverted but this was delayed in some cases, and in the HIV-positive subject required a double dose of vaccine delivered intradermally and subcutaneously. In complex or non-standard PEP delivery, including delayed treatment and immunosuppression due to steroid treatment, HIV or another immunosuppressing medical condition, serology can be used to guide further treatment and should be used to confirm seroconversion.

## Introduction

Without appropriate management, infection with rabies virus or with Australian bat lyssavirus can lead to progressive, fatal neurologic illness. Whilst Australia is free of classical rabies, Australian bat lyssavirus (ABLV) is endemic in local bat populations [1]. Further, Australians are taking increasing numbers of short, return international trips annually, including to regional destinations where rabies is endemic. Many – 64 of 65 individuals requiring post exposure prophylaxis (PEP) in a recent Australian paper [2] – travel without pre-exposure rabies prophylaxis.

National guidelines for PEP of rabies and ABLV, using human rabies immunoglobulin (HRIG) and rabies vaccine, are used in Australia [3], [4]. Reported local exposures to lyssaviruses managed in Queensland are assessed in conjunction with the local Public Health Unit (PHU). The aim of post-exposure vaccination is to achieve an antibody titre of ≥0.5 IU/mL, as per World Health Organization (WHO) guidelines [5], as quickly as possible. In line with the United States [6], Australia moved from a five dose to a four dose standard PEP protocol in November 2010 [7]. Current PEP guidelines for both potential rabies or ABLV contact require that healthy individuals without previous rabies vaccination receive four vaccine doses on days 0, 3, 7, and 14 after exposure, with a fifth dose recommended (day 28) only in the case of immune impairment (through disease or treatment) [4]. Patients who have not undergone pre-exposure prophylaxis receive HRIG as part of PEP to provide early protection against migration of the virus to the central nervous system, until a protective vaccine-induced titre is achieved [8]; usually seen by day 14 [9]. For patients who have received previous rabies vaccination, HRIG is not required and only two doses of vaccine are given on days 0 and 3 [3].

Once commenced, every effort should be made to comply with dosing and timing for PEP schedules, including both HRIG and vaccine. Whilst short interruptions of some days in receiving scheduled doses are generally not of concern, the impact of longer delays of weeks is not known [10]. In these situations, serological testing, to monitor the immune response, taken seven to 14 days following the final vaccine dose in the series, has been recommended [11]. The Australian Immunisation Handbook states that confirmatory serology is not routinely necessary, but should be done two to three weeks following pre-exposure prophylaxis in immunosuppressed patients at risk of exposure to ABLV or rabies, and at two to four weeks following PEP in immunosuppressed patients after the recommended fifth dose given at day 28 [3].

ABLV was first identified in the brain of a young black flying fox (*Pteropus alecto*) found in 1996 unable to fly, and subsequently in a 1995 archived specimen from the same species [12], [13]. ABLV has been identified as the cause in two human deaths, with neither case having received PEP [14], [15]. Bat ABLV seroprevalance is low (<1%) in general surveys, but higher in sick, injured, or rescued bats [1]. Thirty-one (5.2%) of 600 bats submitted to Queensland Scientific Services between 1998 and 2006 were positive for ABLV by direct fluorescent antibody testing [16]. There is no direct empiric evidence demonstrating the effectiveness of current PEP regimens for preventing ABLV in humans. An early study showed mice administered a variety of commercial animal and human rabies vaccines were uniformly protected against intracerebral ABLV challenge [13]. A 2005 study showed 15 of 19 mice vaccinated twice via the intraperitoneal route using a human diploid cell vaccine (HDCV) survived peripheral ABLV exposure, and 10 of 20 survived intracranial exposure [17]. Whilst somewhat reassuring, there are to date no published effectiveness or serology reports on individuals who have received PEP in Australia for potential ABLV exposure.

When potential rabies exposure occurs overseas, the traveller may return home having had an altered or delayed PEP course or not having commenced treatment [2]. Those with a pre-existing medical condition or using medication that results in immunosuppression may also require monitoring of PEP response. Here we present four cases with potential exposure to rabies or ABLV between 2009 and 2011 where treatment deviated from standard recommendations or occurred with concurrent immunosuppression.

## Methods

One potential ABLV and three rabies exposures, non-standard in nature, were referred to Brisbane North PHU for public health physician advice. In this case series, we report PEP schedules and serological responses (Table 1). Vaccination date refers to the number of days post exposure, day 0 being the date of injury/exposure.

**Table 1:** Timing of treatment and serological testing following exposure to rabies virus or Australian Bat Lyssavirus.

Days following injury/exposure	Expected titre (IU/mL)	Patient 1		Patient 2		Patient 3		Patient 4	
		Treatment*	Titre†	Treatment*	Titre†	Treatment*	Titre†	Treatment*	Titre†
0	0	v		v					
1								v+HRIG+corticosteroids	
3	0	v							
4								v+corticosteroids	
6						v+HRIG			
7	rising								
8								v	0.16
9						v			
14	rising					v			
18				vv	0.38			v	0.16
20						v			
22				v	0.44				
26		v							
27			0.31						
28	≥0.50								
29				v	2.17				
30		v							
32								v	3.03
33		v	1.99						
34						v			
36				v					
37									
40		v							
49							<0.12		
63						vv			
98							<0.12		
161						vv‡ (intradermal, subcutaneous)			
175							1.39		

* Treatment abbreviations.

v = single dose of rabies vaccine; vaccine given intramuscularly unless otherwise indicated.

vv = double dose of rabies vaccine.

HRIG = human rabies immunoglobulin.

† Anti-rabies antibody titre EU/mL.

‡ Relative volume of vaccine given intradermally and subcutaneously not available in patient's notes.

Serology specimens were tested by Queensland Health using an ELISA assay, Platelia Rabies II kit (Bio-Rad, Hercules, California, USA), according to manufacturer's guidelines. This assay has very high agreement for detecting total anti-rabies virus glycoprotein antibodies compared to the WHO-recommended standard [18]. Results are expressed in equivalent units per millilitre (EU/mL), which correlate with international units (IU/mL): a value ≥0.50 EU/mL represents seroconversion [18].

In this case series, serum specimens were taken from patients to test for rabies antibodies. Oral informed consent, as opposed to written consent, was provided by patients for these procedures as they were part of routine clinical care. This case series was assembled in retrospect by the physicians who participated in the management of these patients. As such, none of the interventions or management were part of a pre-designed research study which would require prior ethics committee approval. We have written consent from each of the four patients for the inclusion of their de-identified details and clinical story in the case series.

## Results

**Patient 1**, a 62-year-old male traveller, suffered an unprovoked dog bite (rabies status unknown) to his thigh in Thailand in February 2009. The wound was cleaned with soap and water for at least five minutes. On the same day, he received an intramuscular dose of cell-culture derived inactivated rabies vaccine (Verorab, Sanofi Pasteur), but no HRIG.

On day 3 he received a second dose of vaccine in Thailand, but had no further treatment until he sought medical care on day 26, several days after returning to Australia. At this point he was given a third dose of rabies vaccine (Rabipur, CSL Biotherapies/Novartis Vaccines).

Rabies serology titre was collected 24 hours later: this was prior to completion of the standard four dose course, and returned a value of 0.31 EU/mL. After consultation with a public health physician, he was offered a dose of rabies vaccine on the day that the serology became available (day 30), a further dose of rabies vaccine on day 33, with repeat serology and a final dose of rabies vaccine on day 40. Serology collected on day 33 was 1.99 EU/mL. This gentleman received a sixth dose of vaccine on day 40, despite having adequate immunity on day 33 and not in keeping with the five-vaccine protocol in use at the time [3]. The patient remains well.

**Patient 2**, a 25-year-old male, was bitten on the left ring finger by a stray puppy (rabies status unknown) in Thailand in January 2010. The wound was cleaned with soap and water for at least five minutes. He received an intramuscular dose of rabies vaccine in Thailand on the same day, but no HRIG. Shortly afterwards, he returned to Australia but did not seek further treatment until day 18. His general practitioner contacted the local PHU and was asked to perform rabies serology prior to giving two doses of intramuscular rabies vaccine simultaneously, meaning both testing and treatment were not consistent with standard recommendations. The rabies titre result was 0.38 EU/mL. Dose four of rabies vaccine was given and further serology performed (day 22) which gave a titre of 0.44 EU/mL. Further doses of rabies vaccine were given on days 29 and 36. Repeat serology (day 29, not available on day 36) showed a rabies titre of 2.17 EU/mL, and the patient remains well.

**Patient 3**, a 62-year-old male, suffered an unprovoked dog bite (rabies status unknown) on the back of his left calf on a Thai beach in October 2010. The patient did not report wound cleaning. The patient was HIV-positive and had non-insulin dependent diabetes mellitus. His most recent CD4 count, taken approximately six months earlier, was 560/µL (within normal limits). The patient sought care in Thailand six days after the event, where he was given a dose of rabies vaccine (Verorab) and HRIG, and received a second dose of vaccine at the same clinic three days later. On his return to Australia, he presented to his local emergency department (ED) on day 14 where he was given an intramuscular dose of vaccine (Rabipur). He had further doses on days 20 and 34. In view of his HIV status, rabies serology was performed on day 49, which showed a sub-therapeutic titre of <0.12 EU/mL.

At this stage, in consultation with the PHU, an infectious diseases physician and a HIV specialist, he was given a double dose of intramuscular Rabipur immediately (day 63) and serology was repeated four weeks later (titre <0.12 EU/mL).

On day 161 a double dose of vaccine (HDCV, Sanofi Pasteur) was given intradermally and subcutaneously (relative volumes given intradermally and subcutaneously not available from clinical notes). Serology performed two weeks later provided a titre of 1.39 EU/mL. The patient remains well.

**Patient 4**, a 23-year-old male with a medical history that included eczema, asthma, and allergies (eggs, dairy products, some food colourings), was scratched on the back of his neck by a bat while standing under a tree in Brisbane, Queensland, in April 2010. Bat urine also entered his eye. The patient did not report wound cleaning. The bat flew away and was unavailable for identification and ABLV testing. He attended the local ED a few hours after injury, and was advised at that time to return the next morning. HRIG and vaccination (Rabipur) were administered simultaneously the following day, and ten minutes later he developed a generalised rash, facial oedema and tingling in his throat. He was treated for urticaria and allergic angio-oedema with prednisolone (50 mg p.o), promethazine (25 mg IM), and ranitidine (300 mg p.o.). He was discharged on the same day with a prescription for two further daily doses of 50 mg prednisolone. After consultation between ED and PHU, the remaining doses of rabies vaccine were given in ED. In light of the patient's egg allergy, the brand of vaccine was changed to an inactivated rabies vaccine which does not contain traces of egg protein (HDCV, Sanofi Pasteur), for doses on days 4, 8, 18, and 32. Because steroids were co-administered, serology was performed to monitor rabies antibody titres. On days 8 and 18, his titre was 0.16 EU/mL, but had increased to 3.03 EU/mL on day 32. A fifth dose of vaccine was administered to this case in keeping with national guidelines, requiring a five dose PEP regimen following ABLV exposure, at the time [3]. The patient remains well.

## Discussion

Failure of rabies PEP regime has been documented [19], [20], but is uncommon if properly administered [21]. As illustrated by our case series, PEP is often complicated by exposures in tourist settings where reliable prophylaxis may not be available, treatment being delayed until return home, or where variations in administration exist [2], [8]. Delays in starting PEP and not receiving the recommended full course of HRIG and vaccines is common in Australian travellers [2]. These cases also illustrate the importance of timely prophylaxis after a potential rabies/ABLV exposure to ensure an adequate immune response, particularly in the context of medical conditions or treatment that may result in immunosuppression.

None of the individuals in this case series had previously received rabies vaccine. Pre-exposure prophylaxis is recommended for Australian travellers spending more than a month, or working with mammals, in a rabies-endemic region; those likely to receive bites or scratches from Australian bats; or are laboratory personnel working with live lyssaviruses [3]. A recent Australian study questioned the adequacy of these recommendations given that, in their case series, most injuries occurred within 30 days of arrival in a rabies-endemic region, most were injured whilst participating in common tourist activities, more than a third did not initiate contact with animals, and the most common injury sites were hands and fingers – high risk sites for rabies transmission due to rich nerve supply [2]. These findings are reinforced by the pattern of exposure in the three subjects in our case series who were bitten by a dog in Thailand. The authors recommended all travellers to rabies endemic regions be counselled about high-risk behaviours, avoiding animal bites, and be offered pre-exposure vaccination [2].

In each of the cases described, there was a delay between injury and seroconversion. This is particularly important given the incubation period for rabies/ABLV may be as short as 2 weeks, or, rarely, several days [22], [23]. Most rabies deaths occur when there is deviation from WHO PEP guidelines [21], reinforcing the importance of monitoring the serological response to treatment in certain patients, such as those presented above. Patient 1 and 2 received no HRIG as part of their PEP. This is concerning, as rabies has been documented where HRIG has not been infiltrated into the wounds of exposed patients [24], [25]. Failure to access HRIG whilst overseas, particularly in developing countries, appears to be a common problem, even following severe hand or facial injuries [2], [26], [27]. Given the recent shift to a standard four dose PEP schedule, it is encouraging to note that all but one case seroconverted after four doses of rabies vaccine, despite disruptions to recommended timing.

There were a number of deviations from standard testing and treatment in our cases. These testing deviations in this case series have provided serological data at non-standard time points. Both patient 1 and 2 had serology collected before receiving four doses of vaccine, and, as may be anticipated, had not seroconverted. However, these assays showed the immune response in both patients had risen above baseline. Patient 2 received a double dose of vaccine following a prolonged delay after dose 1, and both patients 1 and 2 received six doses of vaccine in total. These events are likely an outcome of excessive caution and confusion that surrounds prolonged deviations from recommended PEP schedules. The delay in patient 3 between his last intramuscular dose (day 63) and effective intradermal/subcutaneous dosing (day 161) was due to discussions about the most appropriate management plan, in the absence of strong evidence, and communicating this with the patient. Rabies is an almost invariably fatal disease meaning uncertainty about PEP implementation generates much anxiety for both patients and clinicians. Deviating from existing guidelines, particularly with early serology or extra vaccine doses, is likely to be inefficient and potentially confusing for the patient and others involved in their care. Every effort should be made to comply with recommended testing and treatment protocols following potential ABLV- or rabies-prone injury.

Patient 4 in our series received short course oral steroids immediately following his first vaccine, and had a protective titre demonstrated on day 32, 14 days after his fourth dose. Of note, short-course oral corticosteroids equivalent to a prednisolone dose of less than 20 mg/day are not thought to interfere with the immune response to vaccination [28]. This patient took 50 mg/day for three days, but had adequate seroconversion two weeks after dose 4.

Failure of pre-exposure prophylaxis and PEP has been documented in individuals with immune deficiency secondary to HIV infection [29], [30]. Patient 3 had well controlled HIV, but had not mounted a protective antibody response 161 days after seven IM doses, despite a recent normal CD4 count. Interestingly, he developed a titre of 1.39 EU/mL shortly after a double dose of HDCV vaccine, given in part intradermally and subcutaneously. There are very limited data about using intradermal rabies vaccine in HIV-infected patients as PEP [31], and whether it may be more effective at producing an immune response in HIV-infected patients than IM vaccine remains unclear. Care is needed in assessing HIV patients after potential rabies exposure even when immunosupression is thought to be mild. Further clinical trials are required to identify optimal PEP regimens in HIV-infected patient groups with varying immunosuppression.

In conclusion, clinicians should be aware that a delay between injury/exposure and PEP administration, schedule interruption, or complicating immunosuppression may inhibit or delay seroconversion. Due to the potentially short incubation period of rabies/ABLV, particular care must be taken in such patients where HRIG has not been administered. In cases with delayed treatment, complicating medical history, or concurrent steroid or other immunosuppressing therapy or medical condition, serology should be used, where available, to monitor the response to PEP and confirm serconversion has occurred.
